# Is neck pain a marker for something serious? Like myelopathy

**DOI:** 10.1038/s41393-024-01041-1

**Published:** 2024-10-14

**Authors:** Alisha W. Sial, Stone Sima, Rajpal Narulla, Nashwa Najib, Mark Davies, Ashish D. Diwan

**Affiliations:** 1 Spine Labs, St George and Sutherland Clinical School, University of New South Wales, Sydney, NSW Australia; 2 Spine Service, Department of Orthopaedic Surgery, St George and Sutherland Clinical School, University of New South Wales, Sydney, NSW Australia; 3 Spinal Surgery, Discipline of Orthopaedic Surgery, School of Medicine, University of Adelaide, Adelaide, SA Australia; 4 Department of Neurosurgery, St George and Sutherland Clinical School, University of New South Wales, Sydney, NSW Australia

**Keywords:** Spinal cord diseases, Chronic pain

## Abstract

Degenerative Cervical Myelopathy (DCM) is a chronic progressive condition of the cervical spine that leads to compression of the spinal cord. It is the most common cause of spinal cord dysfunction in adults, and it occurs due to age-related changes or genetically associated pathologies. DCM is a clinical and radiological diagnosis and presents with a spectrum of symptoms ranging from neck pain and stiffness to paralysis. While neck pain is prevalent amongst patients attending specialist clinics, its predictive value for DCM is limited. This paper focuses on elucidating the relationship between DCM and chronic neck pain, and we discuss the underlying aetiology and broader neurological implications in the context of the literature. The progression of DCM can be slow and insidious with symptoms worsening gradually over time. Neck pain should not be discounted in the evaluation of DCM.

## Introduction

Degenerative Cervical Myelopathy (DCM) is one of the most frequent causes of spinal cord dysfunction in the elderly and comprises eleven older nomenclatures of the condition (Supplementary 1) [1]. DCM encompasses a variety of age-related and genetically associated pathologies including cervical spondylotic myelopathy, degenerative disc disease, and ligamentous aberrations such as ossification of the posterior longitudinal ligaments [2]. These conditions may exist with or without compression of the spinal cord. As the spinal cord becomes compressed and damaged in DCM, a range of neurological symptoms can develop including chronic pain in the neck or paraesthesia of the upper limbs.

Neck pain is a common clinical presentation with a myriad of aetiologies. Persistent neck pain is believed to be a predictor of DCM. However, the relationship between structural alterations in the cervical spine and symptomatic presentations requires comprehensive understanding, particularly given the implications for diagnosis and treatment. Neck pain can be classified into episodic, inflammatory, referred, psychogenic, and chronic. Chronic neck pain and DCM are two conditions that are putatively related to each other with an unclear temporal relationship.

Neck pain therefore may not simply be a symptom of degenerative change in the structure of the spinal column, but rather a symptom that may herald the future development of DCM. In this perspective article, our objective is to elucidate the correlation between DCM and neck pain, specifically distinguishing between episodic or chronic manifestations.

## Degeneration of the intervertebral disk – as a cause of pain

The intervertebral discs in the cervical spine undergo age-related degeneration, leading to structural changes and clinical symptoms, such as cervical region pain exacerbated by movement. Aging reduces the water content in the nucleus pulposus, subsequent protrusion or extrusion can irritate local neural structures, serving as a potential neurological pain source in the cervical region. Progressive IVD changes lead to an unequal distribution of forces across the endplates, resulting in the remodelling of the adjacent vertebral bodies. These changes include increased anteroposterior length of the vertebrae, decreased intervertebral height, osteophytes, and bone spur formation. IVD degeneration can also have downstream consequences such as ligamentous changes, and cervical alignment adverse alterations including the development of kyphosis, scoliosis, hyperlordosis, or listhesis. All of these changes can individually or collectively be often associated with pain [3]. It is important to note that not everyone with degenerative changes in the spine will experience pain. Some people may have degenerative changes on imaging without any accompanying symptoms. Additionally, pain can be influenced by various factors such as the extent of degeneration, overall health and lifestyle, cultural factors, and individual pain thresholds.

## Ischaemic cord injury – a cause of pain?

The compression of the spinal cord is primarily instigated by degeneration of the spinal column elements including the IVD, facet joints, and alteration of the ligamentous anatomy (Supplementary 2A, 2B). As these structures deteriorate, they lead to spinal cord stenosis by exerting direct pressure on the cord, causing mechanical stress. This mechanical stress instigates a cascade of molecular and cellular events including oxidative stress, ischemia, and neuro-inflammation, ultimately leading to neuronal and glial cell death. Wilson et al. describe the degeneration process at the macrovascular and microvascular levels in a review of the latest advances in the management of DCM. At the macrovascular level, degenerative changes to the cervical spine compress the lumen of major feeding arteries, including the vertebral and anterior spinal arteries. At the microvascular level, compression and deformation of the spinal cord led to the eventual loss of penetrating branches of the lateral pial arterial plexus causing the dysfunction of endothelial cells due to ischemia, therefore compromising the blood spinal cord barrier (BSCB). This breakdown of the BSCB can trigger secondary neuro-inflammation, involving macrophage and microglia activation, which causes a rise in inflammatory cells and progressive neuronal and oligodendroglial cell death through apoptotic pathways [4]. The dysfunction of neural elements can also cause pain in conditions such as complex regional pain syndrome (CRPS) [5] and fibromyalgia where there may be less obvious mechanical causes for pain [6].

The pro-inflammatory response due to degeneration of the spinal cord in patients with DCM can lead to significant physiologic changes that potentiate axial neck pain. Chronic oedema and fibrotic changes to the spinal cord can increase sensitivity of the nerves to pain. DCM can lead to chronic inflammation and cellular stress which results in the proliferation of pro-inflammatory cytokines like c-reactive protein, interleukin 6 (IL-6), IL-1 and tumour necrosis factor alpha (TNF-a), which have all been shown to be elevated in patients with neck pain [7]. A decreased creatinine level has also been observed in patients with chronic damage to the spinal cord due to degeneration of the cervical muscles and atrophy from denervation and reduced physical activity, which is a known contributor to neck pain [7, 8]. Mechanoreceptors and chemoreceptors in the cervical musculature can also respond to degenerative changes in the spinal column in patients with DCM, potentiating pain. Unencapsulated nerve free endings in the muscle may respond to the pro-inflammatory biomarkers released by injury or ischemia of the spinal cord. Mechanoreceptors can respond to stretch or pressure in patients with hypermobility and dynamic compression of the spinal cord [9].

## Structural changes

The degenerative changes seen in DCM, overlap with osteoarthritis and changes in the intervertebral discs (IVD), uncovertebral, and facet joints. Capsular ligaments in the cervical spine, which are integral to spinal stability, can become lax over time due to repetitive stress, potentially causing cervical instability [10]. Demonstrated evidence of capsular laxity in the context of DCM is lacking in the literature. It is yet to be determined whether it is stiffness of the cervical spine joints with increasing age or the laxity of the cervical spine ligaments that contribute to people becoming myelopathic (Supplementary 2C). The pathogenesis of myelopathy in patients with DCM can be attributed to three main components:

1. Static factors: processes that lead to cervical canal stenosis and consequent cord compression.
  Acquired spondylosis of the disc, facet vertebral bodies, and ligaments and subsequent loss of lordotic curvature.
  Congenital cervical stenosis (<13 mm in diameter is considered stenotic).
  Ossification of posterior longitudinal ligament.
  Ossification of the ligamentum flavum.
2. Dynamic factors: resulting in cervical cord injury, can be multifactorial.
  Movements of a severely compressed cord are restricted.
  Flexion of the spine results in overstretching of the cord. This effect is more severe in the setting of a prominent ventral osteophyte complex and kyphotic deformity [11].
  Extension causes posterior cord compression by buckling of the ligamentum flavum and shingling of the laminae.
3. Ischemia: Spondylotic cord compression causes the pathologic butterfly pattern of cord ischemia which affects the grey and medial white matter. This pattern is also consistent with a vascular hypoperfusion injury. Compression affects the flow in the small pial and intermedullary arterioles, as well as the larger anterior spinal artery. Venous congestion may also play a role, with the potential for venous infarction to occur. Collectively these processes can result in cavitation within the spinal cord, known as syrinx formation.

## DCM as a spectrum

Neck pain may exist within the entire spectrum of DCM. Patients may initially present to a clinician with simple neck pain or stiffness, and as the condition progresses, patients may experience gait ataxia, autonomic dysfunction, limb weakness, and severe neurological impairment. The physical examination of these patients may reveal signs indicative of upper motor neuron disease, suggestive of spinal cord pathology. A common way of functionally grading DCM patients is with the modified Japanese Orthopaedics Association (mJOA) score. The mJOA score is a specialised clinical tool used to evaluate the severity of neurological deficits in patients with cervical myelopathy. The mJOA assesses motor function in the extremities, sensory function in the upper extremities and trunk, and bladder function. The total score ranges from 0 to 18 with lower scores indicating a more severe neurological impairment. Interpretation of the mJOA score in conjunction with clinical assessment and radiological findings is useful for the diagnosis of cervical myelopathy. Neck pain is not scored in mJOA. Clinicians can alternately use the Neck Disability Index (NDI), a self-reported questionnaire designed to measure neck specific disability by assessing the impact of neck pain on a patient’s daily life.

Neck pain is not always a major symptom but is often an associated symptom of DCM. Neck pain is reported to be as high as 80% in patients with DCM compared with the total population prevalence of neck pain at approximately 15% [12]. It is not known how neck pain is initiated, whether the cause is degeneration, inflammation, or movement. Within the spectrum of DCM, a critical gap remains in our understanding of the dynamic progression of clinical symptoms. Specifically, there is a lack of clarity regarding the fluctuating nature of symptomatology, including the phenomenon of waxing and waning symptoms, the presence of an unstable plateau phase, periods of clinical improvement, and eventual clinical deterioration. The prevalence and progression patterns along with risk factors are critical for the management of asymptomatic cervical cord compression (Supplementary 1). Symptom severity in DCM spans a broad spectrum, complicating the clinical identification of the onset of myelopathy.

The severity of DCM can vary depending on the extent of the damage to the spinal cord and the location of compression. Some individuals may experience only mild symptoms that do not significantly impact their daily lives, while others may experience significant disability and may require surgery. Not all of the clinical manifestations present as pain. Whilst the etiological factors for non-specific neck pain are poorly understood, neck pain correlates with spinal degeneration such as cervical spondylosis [13], however, in the context of DCM, neck pain is a confounder.

## Is there a role of surgery for neck pain

Laminoplasty aims to alleviate mechanical compression of the spinal cord in patients with degenerative cervical myelopathy (DCM), potentially leading to reversible changes that could reduce inflammation and improve cord reperfusion, thereby alleviating neck pain. However, studies have demonstrated that while laminoplasty does not exacerbate neck pain, it also does not significantly improve it [14]. This lack of improvement may be attributed to the patients in these studies having a lower baseline prevalence of neck pain or presenting with spondylotic changes. In patients exhibiting significant spondylotic alterations, DCM, and factors contributing to dynamic spinal cord compression, laminoplasty alone may be insufficient for addressing axial neck pain. In such cases, alternative surgical interventions, such as laminectomy combined with fusion or a hybrid construct involving both anterior and posterior approaches for indirect decompression, might be necessary to relieve spinal cord pressure and mitigate other pain generators in the neck [15]. Further research is essential to elucidate the inflammatory response in patients with DCM and axial neck pain following surgical intervention. Advanced studies employing proteomics and metabolomics are needed to identify potential causal relationships between these factors.

## Conclusion

The pathophysiology of pain in DCM is multifactorial. The nonspecific nature of neck pain may lead clinicians to underestimate DCM, attributing the discomfort to less serious conditions. In the realm of evaluating DCM, a notable discrepancy exists in the assessment of neck pain within existing clinical scales. The mJOA notably omits neck pain as a parameter which necessitates reliance on NDI to gauge the impact of neck pain. Integrating the assessment of neck pain into the standard evaluation process could potentially enhance the understanding of the natural history of DCM and guide clinician assessment and management.

## Supplementary information

Supplementary 1 – Comparing characteristics of different types of degenerative cervical myelopathy (DCM) and altered cord signal change

Supplementary 2 – X-ray and magnetic resonance imaging (MRI) of a 68yo with neck and bilateral shoulder pain and corresponding risk factors for development of degenerative cervical myelopathy. (A) Lat
