# Supplementary material for: Is neck pain a marker for something serious? Like myelopathy
Source: Spinal Cord. 2024 Oct 14;62(12):718–20. doi: 10.1038/s41393-024-01041-1 (PMC11621022; doi:10.1038/s41393-024-01041-1)
Supplement: Supplementary file 1 — Supplementary 1 – Comparing characteristics of different types of degenerative cervical myelopathy (DCM) and altered cord signal change [file 41393_2024_1041_MOESM1_ESM.docx]

**Supplementary 1**

Supplementary 1 – Comparing characteristics of different types of degenerative cervical myelopathy (DCM) and altered cord signal change.

| *Type of Cervical Myelopathy/ condition* | *Definition* | *Aetiology* | *Common features* |
| --- | --- | --- | --- |
| *Classical Degenerative Cervical Myelopathy (DCM)* | A type of spinal cord disorder caused by degenerative changes in the cervical spine, leading to compression of the spinal cord. | Age-related degeneration of cervical spine structures (e.g. intervertebral disc, facet joints etc.) | Compression of the spinal cord resulting in various neurological symptoms (e.g. weakness, numbness, gait disturbance, loss of dexterity). |
| *Stenotic Degenerative Cervical Myelopathy* | A subtype of DCM caused by spinal canal stenosis resulting in compression of the spinal cord. | Age-related degeneration of the cervical spine structures leading to spinal canal narrowing. | Compression of the spinal cord resulting in neurological symptoms. |
| *Non-Stenotic Degenerative Cervical Myelopathy* | A subtype of DCM caused by degenerative changes in the cervical spine without spinal canal stenosis. | Age-related degeneration of the cervical spine structures, such as intervertebral disc degeneration and osteophyte formation. | Compression of the spinal cord resulting in neurological symptoms. |
| *Non-Degenerative Cervical Myelopathy* | A type of cervical myelopathy caused by non-degenerative spinal cord compression such as a tumour or infection. | Tumour, infection, or congenital spinal cord malformation. | Compression of the spinal cord resulting in neurological symptoms. |
| *Spinal Cord Signal Change (SCSC)* | A radiological finding of abnormal signal intensity on the spinal cord MRI, often associated with spinal cord pathology. | Various aetiologies including ischemia, demyelination, and inflammation. | MRI findings of hyperintensity or hypointensity of the spinal cord on T1- or T2-weighted images, respectively. |

It is worth noting that classical and stenotic DCM are not necessarily mutually exclusive, some patients might exhibit features of both types. Additionally, the severity of symptoms and their response to treatment can vary widely between individuals.
