# Supplementary material for: Is neck pain a marker for something serious? Like myelopathy
Source: Spinal Cord. 2024 Oct 14;62(12):718–20. doi: 10.1038/s41393-024-01041-1 (PMC11621022; doi:10.1038/s41393-024-01041-1)
Supplement: Supplementary file 2 — Supplementary 2 – X-ray and magnetic resonance imaging (MRI) of a 68yo with neck and bilateral shoulder pain and corresponding risk factors for development of degenerative cervical myelopathy. (A) Lat [file 41393_2024_1041_MOESM2_ESM.docx]

**Supplementary 2**

**
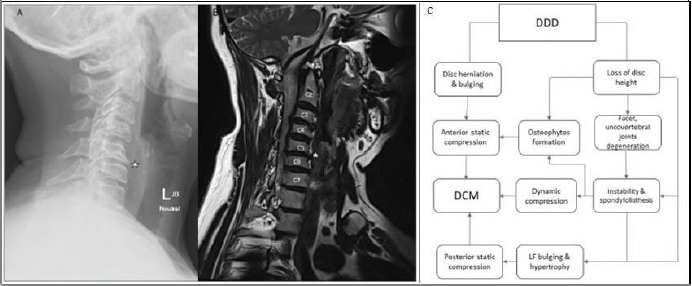
**

B

C

A

Supplementary 2 – X-ray and magnetic resonance imaging (MRI) of a 68yo with neck and bilateral shoulder pain and corresponding risk factors for development of degenerative cervical myelopathy. (A) Lateral X-Ray of the cervical spine shows narrowing of the spinal canal with features of facet joint arthritis. (B) MRI C-spine T2WI showing degeneration from C3-C7 with no canal stenosis or cord compression. At C5-6 there is a broad-based disc bulging with an osteophyte ridge (Star). (C) describes the pathophysiology and risk factors for the development of DCM in the context of degeneration.
